# Weight bias among students and employees in university settings: an exploratory study

**DOI:** 10.1186/s12889-025-21922-1

**Published:** 2025-02-19

**Authors:** Léonie Sohier, Claudia Mc Brearty, Stéphanie LeBlanc, Dominic J. Chartrand, Audrey St-Laurent, Schohraya Spahis, Léonel Philibert, Inès Auclair Mangliar, Marie-Pierre Gagnon-Girouard, Clara Lakritz, Sylvain Iceta

**Affiliations:** 1 School of Psychology, Université Laval, Québec, QC Canada; 2 Research Center of the Quebec Heart and Lung Institute, 2725 Ch Ste-Foy, Québec, QC G1V4G5 Canada; 3 CHU de Québec-Université Laval Research Center, Québec, QC Canada; 4 Department of Medicine, Université Laval, Québec, QC Canada; 5 Department of Kinesiology, Université Laval, Québec, QC Canada; 6 School of Nutrition, Université Laval, Québec, QC Canada; 7 Biochemistry and Molecular Medicine, Université de Montréal, Montréal, QC Canada; 8 Pôle pluralité humaine, Université de l’Ontario Français, Toronto, ON Canada; 9 Department of Psychology, Université du Québec à Trois-Rivières, Trois-Rivières, QC Canada; 10 Department of Psychiatry and Neurosciences, Université Laval, Québec, QC Canada

**Keywords:** Weight bias, Prejudices, Students, Employees, Obesity, University

## Abstract

**Background:**

Weight bias and stigmatization are highly prevalent in modern society, especially in educational settings, such as universities. Despite extensive documentation of the adverse consequences on students’ daily functioning and psychological health, there is limited literature regarding factors associated with weight bias and its extent in Quebec universities.

**Objectives:**

This exploratory study aims to assess the prevalence of weight bias and experiences of weight-related stigmatization, as well as to examine their associations with gender, psychological health problems, and status (students or employees) in a college environment in the province of Quebec.

**Methods:**

Participants were recruited via their university emails. A total of 292 students and 129 university employees participated in an online survey distributed via the secure REDCap platform. The following data was collected: sociodemographic information, status (students or employees), body weight, experiences of stigma, and prejudice towards people living with a higher weight (Fat Phobia Scale; FPS).

**Results:**

Approximately half of the respondents reported experiencing weight-related stigma (44.7%), and half indicated holding prejudice towards overweight people (51.1%), with a moderate rate of bias according to the FPS (3.25). Experience of weight-related stigma was found to be associated with gender (*X*^2^ = 7.88, *p* = 0.019), and a higher prevalence of psychological health problems (*X*^2^ = 9.41, *p* = 0.002), while having prejudice was associated with gender, with men scoring higher at the FPS (F = 7.64, *p* = 0.006), but not with the status (student or employee). The regression model identified significant effects of status [F(4, 347) = 2.856, *p* = 0.005] and the interaction between gender and status [F(4, 347) = -2.326, *p* = 0.021] on the FPS scores.

**Conclusions:**

Various factors are associated with the experience of weight bias and stigmatization towards people with higher weight in the college population. Campaigns to prevent and reduce weight-related bias should be aimed specifically at staff members as well as students. Future research should examine weight bias internalization as a mediator between self-perceived weight and prejudice.

## Introduction

Weight stigma is defined as “the social devaluation and denigration of people perceived to carry excess weight and lead to prejudice, negative stereotyping and discrimination toward those people” [1]. Considered more socially acceptable than many other types of stigmatization [2], this form is omnipresent in various contexts, including the healthcare system [3] and within social settings, such as within families [4]. Weight bias can be defined as the negative attitudes, opinions, and associations that people may have toward individuals living with excess weight. The university setting is not free of weight biases, which have several adverse consequences [5–9]. Among college students, experiencing weight bias is associated with physical activity avoidance, internalized weight stigma, body dissatisfaction, negative affect, exclusion from classroom participation, unhealthful eating, and binge-drinking [6, 7, 9, 10].

Experiencing weight-related bias is also associated with more significant emotional distress, heightened weight concerns, increased risk of eating disorders and moderate to severe anxiety, as well as depressive symptoms [5, 8]. Notably, 64% of students experiencing weight-related bias meet the criteria for at least one mental health disorder (e.g. anxiety disorder and depressive disorder). Even in the absence of stigmatization, the anticipation of bias towards body weight appears to be a crucial factor contributing to psychological health problems. Those psychological health problems are, in turn, associated with weight-related stigmatization [11].

In addition to experiences of weight-related bias, the intensity of those biases that individuals express is also essential to consider. While some studies showed that sociodemographic characteristics and experience of mental health difficulties, like anxiety and depression, are not related to the rate of weight bias [12], other studies tend to demonstrate some associations. Among students and in the general population, women, ethnic minorities, older adults, more educated individuals, and people with a higher weight seem to have fewer weight biases [13, 14]. However, these associations have rarely been explored, and most studies conducted in academic settings have focused on pre-college academic levels, leaving those factors understudied among college students [15]. Nonetheless, evidence indicates that weight-related stigma experienced during university is associated with poorer academic outcomes among students with a higher weight [16].

The educational environment is pivotal in preventing and reducing weight-related stigma [17, 18]. However, according to Nutter and al. (2019)’s literature review, academic settings are often characterized by high levels of weight-related stigma, resulting in significant physical and mental health consequences and poorer self-perceived competence among students. Educators tend to regard students living with a higher weight differently and perceive them as challenging while sharing weight-related biases [15, 19]. As figures of intellectual authority, university professors contribute to shaping social attitudes. In perpetuating or neglecting to challenge weight-related bias, they risk legitimizing these biases, thereby influencing their students’ future social and professional relations [18, 20, 21]. Conversely, sensitized professors and academic employees can play an active role in dismantling these weight-related biases [18, 20]. While most studies on the subject focus on students [15], it is imperative to explore the level of weight-related bias among academic employees since they are an important component in preventing weight-related stigmatization.

Although the scientific literature thoroughly documents the consequences of weight stigmatization, further research into the contributing factors within college settings is essential to develop effective preventive strategies, mitigate its impacts, and implement targeted interventions that address its adverse effects on the academic environment [5, 12, 22]. By examining their practices, universities can enhance their internal policies and increase awareness among students and society about weight-related prejudice and discrimination, thereby aiding in broader cultural change [21]. Consequently, exploring the level of weight-related bias in college settings is essential. To our knowledge, no study has yet examined the factors associated with the level of weight-related bias in college, exploring both students and academic employees.

The overall aim of this study is to explore the level of weight bias among college students and academic employees in a Quebec university setting. More specifically, this research evaluates the association between weight-related bias, both experienced and expressed, and the following factors known to be associated with weight bias: [1] psychological health [2], gender and [3] experience of weight bias. Additionally, the study investigates the differences in weight-related biases between students and employees.

## Methods

### Subjects

Participants eligible for this study included all current students and employees of Université Laval, a francophone university in Quebec, Canada, who were 18 years or older and fluent in French.

### Procedure

A first email was sent to all students and employees via the university’s distribution list for recruitment in May 2022, and a second in June 2022. This email included a hyperlink directing to the secure REDCap (Research Electronic Data Capture) platform [23], where potential participants could access a detailed presentation of the study, the consent form, and the survey. The questionnaire contained sociodemographic and clinical questions, as well as items about bias and beliefs about overweight and obesity. Participants were informed that completing and returning the questionnaires attested to their implicit consent to participate in this research. As the survey contained no direct or indirect identifiers, it was carried out anonymously, and no participant login information was recorded. This study was approved by the ethics committee of Université Laval (*Comité d’éthique de la recherche avec des êtres humains de l’Université Laval*; 2022-075/31-03-2022).

### Measures

#### Sociodemographic and clinical questionnaire

Participants were asked to provide information on the following variables: university status (student or employee), discipline of study or profession, study level for students, age, gender, first language, and visible minority status. Also, they were asked if they had ever received a psychological or physical health diagnosis in their lifetime with yes or no questions.

#### Questionnaire on body weight and stigmatization of overweight and obesity

Participants were asked to provide their weight and height. Also, they were questioned about previous weight loss attempts throughout their lifetime, perceptions of their weight, discomfort with their weight, and if they ever experienced weight-related stigma with yes or no questions. In addition, participants were questioned about the stigmatization of overweight and obesity in the academic environment, including their subjective perception of the level of stigmatization in their academic environment and their opinion of the relevance of a campaign addressing weight stigma, with yes or no questions.

#### Fat phobia scale (FPS)

The short form of the FPS used in this study [24] is a fourteen-item self-report scale designed to measure beliefs and feelings toward people who live with overweight or obesity [25]. This scale assesses perspectives on stereotypical traits related to higher weight. Respondents are invited to evaluate each dichotomous adjective sometimes used to characterize overweight people (e.g. lazy vs. industrious). A higher mean score indicates more weight bias, where the total score ranges between one and five. The internal consistency coefficient of the short form is 0.83 [12]. In this study, the internal consistency of the short version of the FPS is 0.87 for the whole sample, 0.86 for students only, and 0.87 for employees only.

### Statistical analysis

Continuous variables were expressed as mean (M) ± standard deviation (SD), and categorical variables were expressed as count (n) and percentage (%). Continuous variables were compared between genders and status using the student *t*-test or the Mann-Whitney U test, while categorical variables were compared between genders and status using the chi-square test. A P-value of less than 0.05 indicated statistical significance. The Phi measure of association ($\:\varphi\:$) was used to evaluate the strength of relationships between variables of interest. Analyses were conducted using SPSS Statistics Version 29.0.2.0 [26] and were performed on all available data for each item; no data was imputed or removed, although not all participants answered all items.

Correlation analyses were conducted using the Phi coefficient for binary variables and Spearman’s rank correlation for continuous variables. A heatmap was generated to visualize the correlation matrix and explore patterns of association among the variables.

To investigate predictors of FPS scores, multivariate linear regression analyses were performed with the FPS score as the dependent variable. The following independent variables were included in the initial models: age, gender (restricted to “women” and “men,” as the “other” category contained only five participants), Body Mass Index (BMI), employment status (student or employee), history of physical health problems, history of psychological health problems, history of weight-loss attempts, witnessing weight-related stigma (“Have you ever witnessed weight stigma?“), experiencing weight-related stigma (“Have you ever experienced weight stigma?“), and feelings of embarrassment about weight (“Are you embarrassed about your weight?“).

A stepwise model selection procedure was employed to derive the most parsimonious models. This approach combined forward and backward selection, iteratively adding or removing predictors based on the Akaike Information Criterion (AIC). Predictors that minimized the AIC, thus improving model fit, were retained in the final models. The stepwise procedure ensures a balance between explanatory power and model simplicity, reducing the risk of overfitting.

Regression analyses were performed on the full sample to identify general predictors of FPS scores. Additionally, separate models were developed for students and employees to investigate potential subgroup-specific differences in predictors, allowing for a more nuanced understanding of the factors influencing FPS scores within these populations.

## Results

### Population characteristics

The sample included 421 participants, of whom 292 (69.4%) were college students, and 129 (30.6%) were employees from various university departments. The average age of student respondents was 29.2 $\:\pm\:\:$9.2 years old, compared to 41.4 $\:\pm\:\:11.0\:$years old for the employees. Participants’ characteristics are detailed in Table 1.

**Table 1:** Participant characteristics (*n* = 421)

Variables (*n*)	*n* (%)
**Age (** ***n*** ** = 402)**	
30 or less	208 (49.4)
31 to 50	159 (37.8)
51 to 70	35 (8.3)
**Gender (** ***n*** ** = 403)**	
Woman	321 (76.2)
Man	77 (18.3)
Other	5 (1.2)
**Visible minority status (** ***n*** ** = 381)**	
Belongs to a visible minority	30 (7.1)
Not a visible minority	351 (83.4)
**First language (** ***n*** ** = 403)**	
French	370 (87.9)
English	10 (2.4)
Other	23 (5.5)
**University status (** ***n*** ** = 421)**	
Undergraduate student	181 (42.0)
Master’s student	67 (15.9)
Ph.D. student	50 (11.9)
Employees	129 (30.6)
**History of psychological health problems (** ***n*** ** = 390)**	
Yes	94 (22.3)
No	296 (70.3)
**History of physical health problems (** ***n*** ** = 390)**	
Yes	177 (42.0)
No	213 (50.6)
**Body Mass Index (** ***n*** ** = 377)**	
Less than 18.50	11 (2.6)
18.50 to 24.99	136 (32.3)
25.00 to 29.99	65 (15.4)
30.00 to 34.99	59 (14.0)
35.00 to 39.99	52 (12.4)
40.00 or more	54 (12.8)
**Subjective weight interpretation (** ***n*** ** = 368)**	
Healthy	136 (32.3)
Overweight	125 (29.7)
Obesity	98 (23.3)
Don’t know	9 (2.1)
**Attempts to lose weight (** ***n*** ** = 392)**	
1 time	33 (7.8)
2 to 3 times	86 (20.4)
4 times or more	174 (41.3)
Never	98 (23.3)

### Weight-related stigmatization in the university settings

Our results showed that approximately half of the participants (51.3%; *n* = 216) reported feeling embarrassed by their weight, and 44.7% (*n* = 188) reported having experienced weight-related stigma at least once in their lifetime. Additionally, half of the respondents (51.1%; *n* = 215) reported holding prejudices against people with a higher weight, while 36.6% (*n* = 154) did not, according to the yes or no question.

Many respondents (45.8%; *n* = 193) did not witness significant bias towards people with a higher weight in their college setting, yet nearly half (47.7%; *n* = 201) felt that a campaign addressing weight-related bias would be needed.

### Own experience of weight-related bias

#### Gender

The analyses indicated that the prevalence of experienced weight-related bias varied significantly according to gender (*X*^2^ = 7.88, *p* = 0.019), with a small effect size ($\:\varphi\:$ = 0.15). Indeed, 55.3% (*n* = 161) of women reported having experienced stigmatization, compared to 36.4% (*n* = 24) for men and 60.0% (*n* = 5) for other genders.

#### Psychological health

There was also a significant interaction between the prevalence of weight-related bias experienced and the history of psychological health issues (*X*^2^ = 9.41, *p* = 0.002), with a small effect size ($\:\varphi\:$ = 0.16).

#### Student or employee status

The participant’s status as a student or employee was not significantly related to the prevalence of experience of weight-related bias (*p* = 0.876).

### Expressed weight bias towards people with higher weight

#### Subjective perception of their weight

There was no association between their perception of their weight (healthy, overweight, obesity, or don’t know) and if they report having prejudices about weight (*X*^2^ = 6.03, *p* = 0.110).

#### Fat phobia scale (FPS)

Participants’ mean score on the FPS, which measures bias towards people with higher weight, was 3.25 $\:\pm\:$ 0.60, indicating that students and employees held moderate prejudices. Scores ranged from a minimum of 1.71 to a maximum of 4.93. The items with the highest bias rate were that individuals with a higher weight like to eat (M = 4.04 $\:\pm\:\:$0.85), overeat (M = 3.82 $\:\pm\:\:$0.84), and have low self-esteem (M = 3.53 $\:\pm\:\:$0.94).

The results revealed a gender difference on the FPS scores, with men (M = 3.60 $\:\pm\:$ 0.70) scoring significantly higher than women (M = 3.17 $\:\pm\:\:$0.55; F = 7.64, *p* = 0.006).

Furthermore, there was no significant difference in weight bias scores between people who had experienced weight-related bias (M = 3.26 $\:\pm\:$ 0.63) compared to people who did not (M = 3.25 $\:\pm\:$ 0.58; F = 1.19, *p* = 0.276). There was also no significant difference in scores between students (M = 3.29 $\:\pm\:\:$0.60) and employees (M = 3.16 $\:\pm\:$ 0.59; F = 0.73, *p* = 0.393).

### Correlation analyses

**Fig. 1:**
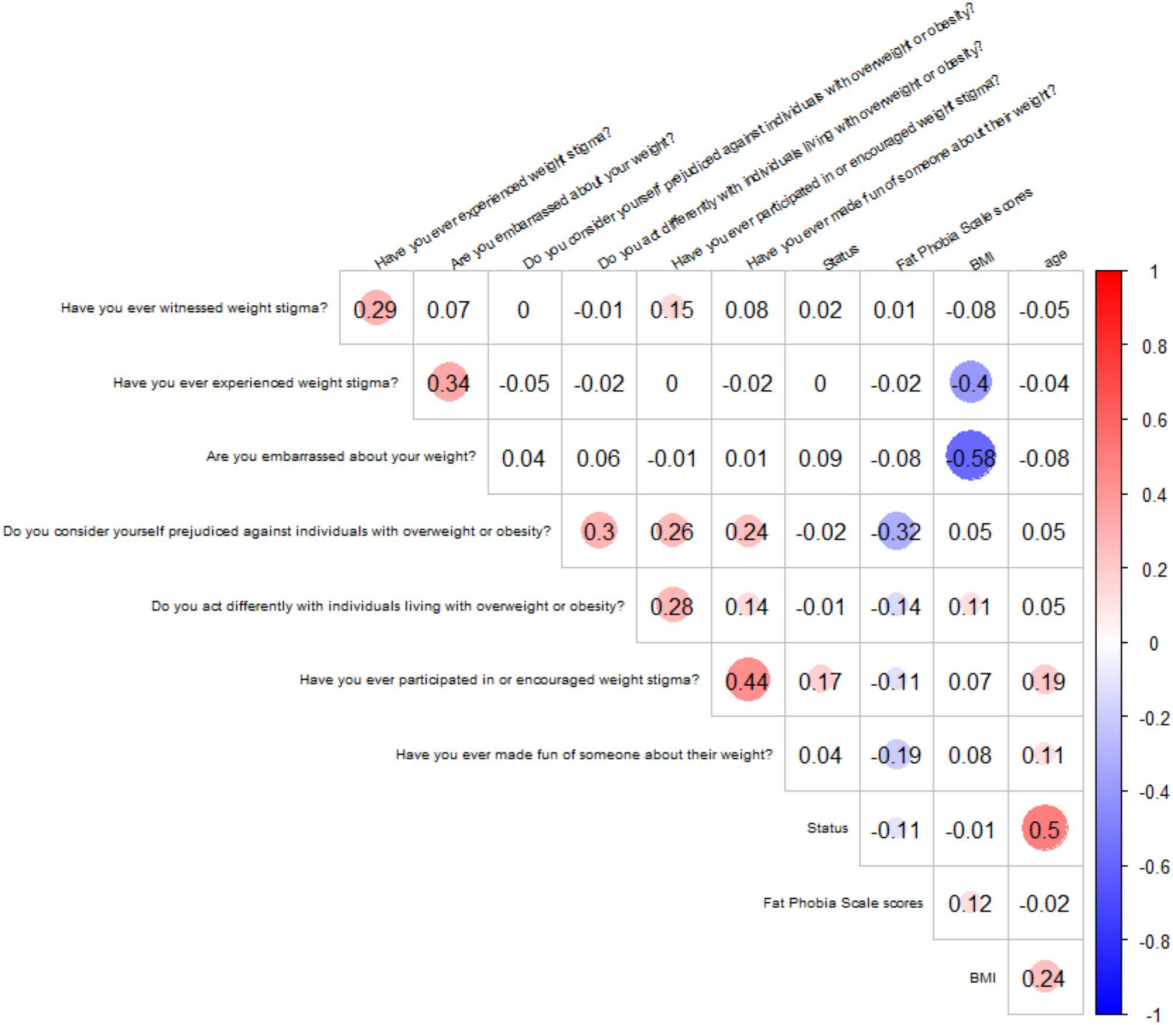
Heatmap of correlation matrix using Phi coefficient for binary variables and Spearman correlation for continuous variables

Results of correlation analyses (see Fig. 1) revealed that the FPS scores did not correlate with BMI or age. However, significant correlations were found between BMI and “Are you embarrassed about your weight?” And between BMI and “Have you ever experienced weight stigma?”.

### Multivariate linear regression analyses

The regression model on the full sample, developed through a stepwise procedure, identified significant effects of status [F(4, 347) = 2.856, *p* = 0.005] and the interaction between gender and status [F(4, 347) = -2.326, *p* = 0.021] on the FPS scores (refer to Table 2). In contrast, gender [F(4, 347) = -0.861, *p* = 0.390] and BMI [F(4, 347) = 1.794, *p* = 0.074] did not demonstrate significant effects, although the p-value for BMI was close to the threshold for significance at *p* = 0.074. The variance explained by the model (adjusted R^2^) was 0.10.

**Table 2:** Multivariate linear regression results predicting Fat Phobia Scale scores among the full sample

	Estimate	Standard error	Statistic	*p* value	95% CI	
					low	high
(Intercept)	3.097	0.169	18.277	0.000	2.764	3.430
Gender[Women]	-0.128	0.149	-0.861	0.390	-0.420	0.164
BMI	0.006	0.003	1.794	0.074	-0.001	0.012
Status[Student]	0.452	0.158	2.856	0.005	0.141	0.763
Gender[Women]*Status[Student]	-0.404	0.174	-2.326	0.021	-0.745	-0.062
	R^2^	adj R^2^	AIC	BIC		
Model	0.11	0.10	601.123	624.304		

CI: Confidence Interval; AIC: Akaike Information Criterion; BIC: Bayesian information criterion; R^2^: variance explained by the model; adj R^2^: variance explained by the model accounted for non-significant predictors

The post-hoc analysis revealed that men students exhibited higher FPS scores than women students [Men students: M = 3.71 ± 0.6; Women students: M = 3.18 ± 0.6, Mann-Whitney U test: U = 7430 *p* < 0.001], and than women employees [Men students: M = 3.71 ± 0.6; Women employees: M = 3.14 ± 0.5; U = 1184, *p* < 0.001], but not men employees [Men students: M = 3.71 ± 0.6; Men employees: M = 3.28 ± 0.9; U = 301, *p* = 0.450].

When conducting regression models on the sample of students (see Table 3), results revealed a significant effect of gender only [F(2,229) = -5.668, *p* < 0.001], women exhibited lower scores than men. The p-value for BMI was again not so far from the threshold for significance at *p* = 0.111. The variance explained by the model (adjusted R^2^) was 0.12.

**Table 3:** Multivariate linear regression results predicting Fat Phobia Scale scores among the student sample

Student only	Estimate	Standard error	Statistic	*p* value	95% CI	
					low	high
(Intercept)	3.518	0.140	25.101	0.000	3.242	3.794
GenderWomen	-0.518	0.091	-5.668	0.000	-0.698	-0.338
BMI	0.006	0.004	1.599	0.111	-0.001	0.014
	R^2^	adj R^2^	AIC	BIC		
Model	0.128	0.121	396.306	410.092		

CI: Confidence Interval; AIC: Akaike Information Criterion; BIC: Bayesian information criterion; R^2^: variance explained by the model; adj R^2^: variance explained by the model accounted for non-significant predictors

Finally, the regression models on the sample of employee did not reveal any effect of the variables, the age was the closest to significance [F(1,104) = 1.785, *p* = 0.077].

## Discussion

The results of this exploratory study have significant implications for the academic community. The study highlights the presence of weight-related bias in the college environment, revealing a moderate level of bias at the FPS (as defined by 27) towards individuals living with higher weights among both students and employees. Our findings also showed that gender and psychological health problems were associated with the experience of weight-related bias. On the other hand, while gender was associated with the level of weight-related bias, the experience of stigmatization, perceptions of their weight, and college status (i.e. student or employee) were not. The results suggest an association between the prevalence rate of mental health problems and the experience of weight-related stigma, in agreement with the literature [5, 11]. Furthermore, according to the regression model, status and the interaction between gender and status have a significant effect on the rate of bias at the FPS for the whole sample, with male students having a higher rate of bias than female students and employees.

According to our findings, the FPS scores showed a lower rate of weight-related bias in a college setting (3.25) when compared to previous studies in the general population (3.6; [24]) and the healthcare setting (3.5; [3]). Since our sample included a high proportion of people enrolled in postgraduate studies, this lower rate of bias compared to other populations is consistent with what has been demonstrated: a higher level of education tends to reduce the level of weight-related bias [28, 29]. Nevertheless, the moderate bias rate aligns with our findings that approximately half of the respondents reported experiencing weight-related bias, and half reported having prejudices towards people with higher weight. These results underscore a major concern for the universities since college settings are supposed to be an environment aimed at preventing weight-related stigmatization and shaping the values of future generations [21]. It is worrying to observe a moderate bias rate, and universities should be committed to reducing these weight-related biases.

The results showed no significant association between participants’ subjective perception of their weight and whether they reported having weight-related prejudice. However, significant positive correlations were obtained between BMI, feelings of embarrassment, and experiences of stigma. This finding suggests that individuals with higher BMI are not only more prone to experience weight stigma but may also internalize these experiences, as evidenced by their heightened embarrassment about weight. Like this study, prior studies have shown mixed results regarding the relationship between weight and the level of weight-related bias [30, 31]. This inconsistency might be explained by the lack of consideration for a potential mediating variable in this association: the internalization of weight stigma. It has been suggested that the internalization of weight stigma fosters negative weight-related stereotypes (e.g. fat people are lazy), thereby increasing the rate of stigmatization [32]. Considering that not all people with a higher weight carry internalized weight stigma, the absence of this mediating factor could account for the mixed results.

Findings from this study indicated that the prevalence of weight-related bias experienced varied significantly according to gender. Regression analyses revealed that the interaction between gender and status was statistically significant, with male students showing higher scores than female students and employees. Similarly, results indicated that gender was the sole significant predictor of FPS scores in the student subgroup. This suggests that being a male student predicts a greater bias toward individuals with higher weight. These results align with previous research showing that males typically hold more negative attitudes toward individuals who are overweight compared to females [33–35]. The milder discriminatory attitude observed among females could be attributed to their increased sensitivity to weight criticism and stigmatization, potentially fostering greater empathy or tolerance toward individuals with higher body weight [14]. Moreover, prior studies have shown that females, particularly those with higher weight, report a higher frequency of bias experiences compared to males [14, 36]. This disparity in weight-related bias is further underscored by the fact that discrimination against females begins at lower proportional weights than males, with females being often judged more severely than males at the same body mass index (BMI; [3, 37]). Consequently, females are more likely to experience weight-related bias, driven by societal norms that emphasize thinness, leading to more severe judgments and adverse treatment compared to their male counterparts [14].

It can be speculated that having previous experiences of weight-related stigma could lead to a lower rate of bias towards others, as supported by studies showing that individuals with family members or friends who have faced such bias tend to exhibit lower rates of bias [13]. Interestingly, this study showed no association between the rate of weight-related bias and personal experiences of this type of bias. It is possible that the lack of precision regarding the self-reported answers in this study explains the absence of a relation. Indeed, participants were asked whether they had ever experienced weight-related stigmatization, a question that encompassed stigmatization associated with both lower and higher weight, as well as varying degrees of severity and frequency. These potentially confounding variables should be further investigated to clarify their effect on the relationship between the bias experienced and exhibited.

No significant difference in weight-related bias was found between employees and students, both displaying moderate levels of prejudice. However, student status predicts higher scores on the FPS. This similarity between groups aligns with a previous longitudinal study that indicated a stable prevalence of weight-based bias over the past decade, reflecting no significant changes in societal norms concerning the acceptability of weight-related bias [38]. Moreover, both students and employees emphasized the importance of a campaign targeting weight-related bias, and the data from this study support this need within the college population, highlighting the need to include both employees and students in future research on weight bias. Targeting employees and teachers in this environment is even more important due to their significant role in shaping students’ social attitudes. Ideally, they should help to reduce weight-related biases rather than exhibiting an equivalent rate of bias as students, thereby legitimizing them [18, 20].

There are some limitations to consider. Firstly, the respondents may not be representative of the overall college population, which jeopardizes the generalizability of results. In fact, participation in the survey was voluntary, which could indicate that participants were interested in the topic of weight-related bias. Also, this study’s sample lacked ethnic and gender diversity. Future research should investigate weight-related bias across a more diverse range of ethnicities and genders. Secondly, weight-related biases are subject to social desirability bias, which may underestimate the true extent of the stigmatization [24]. Future studies could address this limitation by including a social desirability questionnaire to assess and control for this bias in the analyses. Thirdly, the history of mental health problems is based on a self-reported diagnosis. It is known that a significant proportion of the population has clinically significant mental health symptoms without having been diagnosed by a professional [39]. As a result, the prevalence of mental health problems may have been underestimated in this study. Fourthly, several factors were answered by yes or no questions, which reduces the validity of our measures. Validated measurement tools providing more details on all factors would provide a more thorough examination of weight-related biases in the college population. Finally, this study is cross-sectional and does not allow any conclusions to be drawn regarding causality between the factors studied.

## Conclusion

Weight-related bias is prevalent in the college population, with an equivalent rate among students and employees, although academic employees could make a great contribution to reducing weight stigmatization in the social culture. Interventions and campaigns working to reduce weight-related stigma should, consequently, focus more closely on university employees and professors. Gender seemed to be a key factor influencing not only the experience of weight-related bias but also prejudice towards people of higher weight. Further studies are required to determine the reproducibility of these findings in other academic institutions. It would also be beneficial to consider including measures of weight bias internalization in future research, particularly as a potential mediator between self-perceived weight and perceived weight-related prejudice.

## Data Availability

The datasets used and/or analyzed during the current study are available from the corresponding author on reasonable request.

## Abbreviations

BMI: Body Mass Index
FPS: Fat Phobia Scale
M: Mean
REDCap: Research Electronic Data Capture
SD: Standard Deviation
